# Structure cristalline du composé intermétallique Ni_18_Ge_12_

**DOI:** 10.1107/S2056989015003680

**Published:** 2015-02-28

**Authors:** Mohammed Kars, Adrian Gómez Herrero, Thierry Roisnel, Allaoua Rebbah, L. Carlos Otero-Diáz

**Affiliations:** a Université Houari-Boumedienne, Faculté de Chimie, Laboratoire Sciences des matériaux, BP 32 El-Alia 16111 Bab-Ezzouar, Algérie; b Centro de Microscopia Electrónica, Universidad Complutense, 28040 Madrid, Spain; c Centre de Diffractométrie X, Sciences Chimiques de Rennes, UMR 6226 CNRS Université de Rennes 1, Campus de Beaulieu, Avenue du Général Leclerc, France; d Departomento Inorgánica, Facultad C.C. Químicas, Universidad Complutense, 28040 Madrid, Spain

**Keywords:** crystal structure, nickel germanide, inter­metallic compound, B8-type substructure, Ge⋯Ni inter­actions

## Abstract

Single crystals of octa­deca­nickel dodeca­germanide were grown by chemical transport reaction. The inter­metallic compound crystallizes in a superstructure of the hexa­gonal NiAs type (B8 type). All atoms in the asymmetric unit lie on special positions except one Ni atom (two Ni atoms have site symmetry -6.. and another one has site symmetry .2. while the Ge atoms have site symmetries 32., *m*.. and 3..). In the structure, the Ni atoms are arranged in 11- or 13-vertex polyhedra (CN = 11–13). The coordination polyhedra of the Ge atoms are bicapped square anti­prisms (CN = 10) or 11-vertex polyhedra (CN = 11). The structure exhibits strong Ge⋯Ni inter­actions, but no close Ge⋯Ge contacts are observed. The Ni atoms with CN = 13 form infinite chains along [001] with an Ni—Ni distance of 2.491 (2) Å.

## Abstract 2

The title compound was synthesized using solid-state reaction and characterized by X-ray diffraction. The structure crystallizes in an own structure type which is a commensurate superstructure of an underlying B8-type substructure.

## Contexte chimique   

Grâce à leurs excellentes propriétés physiques, à savoir une basse température de formation, une faible résestivité et une stabilité sur une large plage de température, les germaniures de nickel sont des candidats très prometteurs pour de futures applications en microélectroniques comme matériaux de contact (Gaudet *et al.*, 2006 ▸; Husain *et al.*, 2009 ▸; Dellas *et al.*, 2010 ▸). Cet intérêt s’est élargi à la fabrication des couches minces, des nanofils et des nanoparticules à coeur-coquille (Grebenkova *et al.*, 2012 ▸; Yan *et al.*, 2011 ▸; Lai *et al.*, 2014 ▸). Le diagramme de phase ainsi que les propriétés thermodynamiques du système Ge–Ni portent toujours un grand intérêt (Liu *et al.*, 2010 ▸; Jin *et al.*, 2012 ▸). Ce diagramme de phase a été au préalable étudié par plusieurs groupes (Ruttewit & Masing, 1940 ▸; Ellner *et al.*, 1971 ▸; Dayer & Feschotte, 1980 ▸). En effet il existe neuf composés intermétalliques: βNi_3_Ge, γNi_3_Ge, δNi_5_Ge_2_, Ni_2_Ge, ∊Ni_5_Ge_3_, Ni_19_Ge_12_, Ni_3_Ge_2_, ∊′Ni_5_Ge_3_ et NiGe dans le diagramme de phase Ge–Ni revu par Nash & Nash (1987 ▸). Cependant le travail le plus important effectué dans ce système dans la région de composition entre 25–50 at% Ge reste celui d’Ellner *et al.* (1971 ▸), qui a permis de caractériser et d’élucider les structures cristallines de ces phases par diffraction des rayons X. En se basant sur les intensités déduites des films de Weissenberg, Ellner *et al.* (1971 ▸) ont favorisé pour la phase Ni_19_Ge_12_ un modèle de superstructure de symétrie monoclinique (*a* = 11.63, *b* = 6.715, *c*= 10.048 Å et β = 90°); plutôt qu’une symétrie hexa­gonale (*a* = *b* = 6.72 et *c* = 10.05 Å). L’étude par diffraction électronique menée par Larsson & Withers (1998 ▸) sur cette phase montre une superstructure commensurable et confirme seulement une symétrie monoclinique localisée.

## Commentaire structurelle   

**Figure 1:**
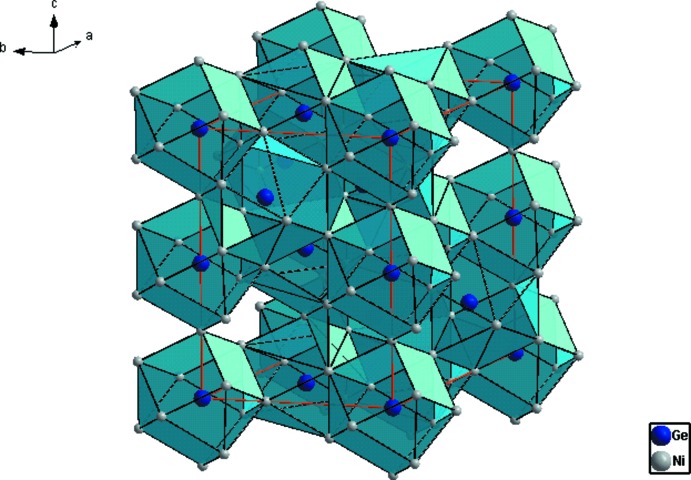
Structure de Ni_18_Ge_12_ montrant l’empilement des polyèdres de coordination des atomes de Ge.

**Figure 2:**
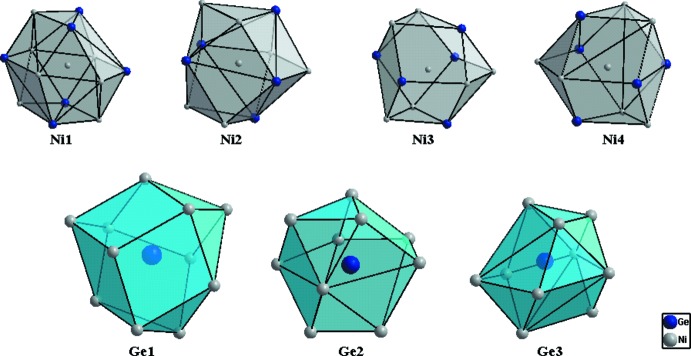
Polyèdres de coordination des atomes Ge et Ni dans la structure de Ni_18_Ge_12_.

**Figure 3:**
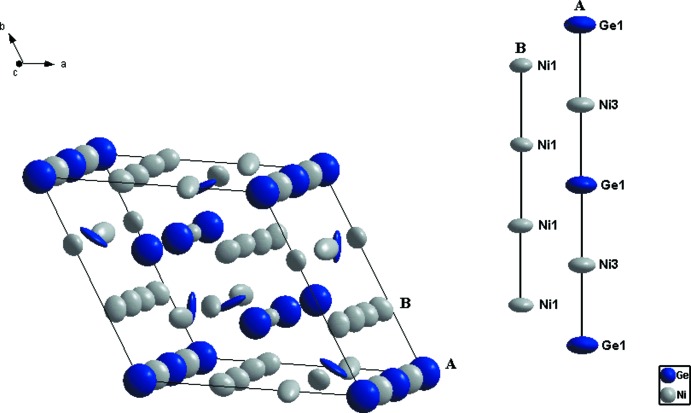
Maille élémentaire de la structure de Ni_18_Ge_12_ montrant la séquence des atomes dans les deux types de chaînes, avec un déplacement des ellipsoïdes à 95% de probabilité.

La phase Ni_18_Ge_12_ qui cristallise dans une superstructure de symétrie hexa­gonale, résulte d’une occupation ordonnée des lacunes d’une simple structure NiAs avec des paramètres de maille doublés (2*a* × 2*a* × 2*c*) et donc un volume de maille huit fois plus important (Fig. 1 ▸). Les atomes de Ni possède une coordination (CN) égale à 11 et sont caractérisés par des prismes trigonal à faces coiffées, à l’exception de l’atome Ni1 dont la coordination est la plus élevée CN = 13. Les atomes de Ge sont eux caractérisés par des anti­prismes carrés à faces coiffées (CN = 10) à l’exception de l’atome Ge1 dont la coordination est égale à 11 (Fig. 2 ▸). Cette coordination n’implique que les atomes de Ni, ainsi les distances de liaisons Ge–Ge ne sont pas observées. Les distances Ge—Ni et Ni—Ni varient entre 2.219 (2)–2.709 (2) Å et 2.491 (2)–2.579 (2) Å, respectivement, et sont comparables à celles observées dans les composés binaires Ni_*x*_Ge_*y*_ (Ge—Ni = 2.12–2.88 Å et Ni—Ni = 2.38–2.81 Å; Pfisterer & Schubert, 1950 ▸; Ellner *et al.*, 1971 ▸; Larsson & Withers, 1998 ▸; Takizawa *et al.*, 2000 ▸); ou observées dans d’autres composés intermétalliques du système Al–Ge–Ni (Ge—Ni = 2.320–2.807 Å et Ni—Ni = 2.502–2.682 Å; Jandl *et al.*, 2013 ▸). Toutefois la distance Ge—Ni est légèrement inférieure à la somme des rayons covalents (2.77 Å) (Emsley, 1989 ▸), ce qui explique une forte inter­action Ge—Ni. Cette structure est caractérisée par la présence de duex2 types de chaînes métalliques de coordonnées (0 0 *z*) et (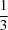 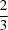 *z*). Les atomes de Ge1 et Ni1 alternent le long de la chaîne (0 0 *z*), le même phénomène a été observé dans la structure Ni_5_As_2_ (Oryshchyn *et al.*, 2011 ▸), alors que la chaîne (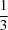 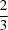 *z*) est seulement caractérisée par un enchaînement d’atomes Ni1 (Fig. 3 ▸), avec des distances Ni1—Ni1 similaires à celle observées dans le Ni métallique (2.49 Å) (Swanson & Tatge, 1953 ▸).

## Synthèse et cristallisation   

Les monocristaux de Ni_18_Ge_12_ ont été obtenus lors des essais de synthèses du clathrate Ge_30_Ni_16_I_8_, à partir d’un mélange d’éléments purs. Le mélange broyé puis scellé dans un tube de quartz est porté à une température de 1073 K pendant dix jours.

## Affinement   

Détails de données crystallines, collection de données et affinement sont résumées dans le tableau 1 ▸. La structure a été affinée dans le groupe d’espace *P* 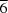
2c sur la base du modèle structural proposé par Ellner *et al.* (1971 ▸), avec une occupation de moitié des atomes Ni3 et Ni4 des sites 2*b* et 6*g*, respectivement. La composition du germaniure obtenue en fin d’affinement Ge_11.868_Ni_18.06_ [Ni(at%) = 60.35; Ge(at%) = 39.65] est proche de celle déduite par analyse chimique MET [Ni(at%) = 60.03; Ge(at%) = 40.07]. L’affinement du paramètre de Flack suggère la présence d’une macle par inversion, la fraction en volume des composants est 0.62 (12): 0.38 (12). En fin d’affinement la carte de densité électronique est de ρ_max_ = 2.02 e Å^−3^ (localisée à 1.06 Å de Ge3) et ρ_min_ = 1.54 e Å^−3^ (localisée à 0.90 Å de Ge2).

## Supplementary Material

Crystal structure: contains datablock(s) global, I. DOI: 10.1107/S2056989015003680/ru2062sup1.cif

Structure factors: contains datablock(s) I. DOI: 10.1107/S2056989015003680/ru2062Isup2.hkl

CCDC reference: 1050846

Additional supporting information:  crystallographic information; 3D view; checkCIF report

## Figures and Tables

**Table 1:** Dtails exprimentaux

Crystal data	
Formule chimique	Ni_18,06_Ge_11,87_
*M*_r_	1921,88
Systme cristallin, groupe d’espace	Hexagonal, *P* 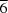 2*c*
Temprature (K)	105
*a*, *c* ()	6,6585(13), 9,962(3)
*V* (^3^)	382,49(15)
*Z*	1
Type de rayonnement	Mo *K*
(mm^1^)	44,48
Taille des cristaux (mm)	0,16 0,10 0,04

Collection de donnes	
Diffractomtre	Bruker APEXII
Correction d’absorption	Multi-scan (*SADABS*; Sheldrick, 2002 ▸)
*T*_min_, *T*_max_	0,047, 0,160
Nombre de rflexions mesures, indpendantes et observes [*I* > 2(*I*)]	3412, 837, 393
*R*_int_	0,107
(sin /)_max_ (^1^)	0,909

Affinement	
*R*[*F* ^2^ > 2(*F* ^2^)], *wR*(*F* ^2^), *S*	0,062, 0,091, 1,26
Nombre de rflexions	837
Nombre de paramtres	35
_max_, _min_ (e ^3^)	2,02, 1,54
Structure absolue	Flack (1983 ▸), 341 paires de Friedel
Paramtre de structure absolue	0,38(12)

Programmes informatiques: *APEX2* et *SAINT* (Bruker, 2006 ▸), *SIR97* (Altomare *et al.*, 1999 ▸), *JANA2000* (Petek *et al.*, 2014 ▸) et *DIAMOND* (Brandenburg Putz, 2009 ▸).

## supplementary crystallographic information

### Crystal data

**Table:**

Ni_18.06_Ge_11.87_	*D*_x_ = 8.339 Mg m^−^^3^
*M**_r_* = 1921.88	Mo *K*α radiation, λ = 0.71073 Å
Hexagonal, *P*62*c*	Cell parameters from 962 reflections
Hall symbol: P -6c -2c	θ = 4.1–39.8°
*a* = 6.6585 (13) Å	µ = 44.48 mm^−^^1^
*c* = 9.962 (3) Å	*T* = 105 K
*V* = 382.49 (15) Å^3^	Plate, black
*Z* = 1	0.16 × 0.10 × 0.04 mm
*F*(000) = 886	

### Data collection

**Table:**

Bruker APEXII diffractometer	837 independent reflections
Radiation source: x-ray tube	393 reflections with *I* > 3σ(*I*)
Graphite monochromator	*R*_int_ = 0.107
CCD rotation images, thin slices scans	θ_max_ = 40.2°, θ_min_ = 3.5°
Absorption correction: multi-scan (*SADABS*; Sheldrick, 2002)	*h* = −11→11
*T*_min_ = 0.047, *T*_max_ = 0.160	*k* = −12→9
3412 measured reflections	*l* = −16→18

### Refinement

**Table:**

Refinement on *F*	Weighting scheme based on measured s.u.'s *w* = 1/(σ^2^(*F*) + 0.0001*F*^2^)
*R*[*F*^2^ > 2σ(*F*^2^)] = 0.062	(Δ/σ)_max_ = 0.0002
*wR*(*F*^2^) = 0.091	Δρ_max_ = 2.02 e Å^−^^3^
*S* = 1.26	Δρ_min_ = −1.54 e Å^−^^3^
837 reflections	Extinction correction: B-C type 1 Gaussian isotropic (Becker & Coppens, 1974)
35 parameters	Extinction coefficient: 200 (40)
0 restraints	Absolute structure: Flack (1983), 341 Friedel pairs
0 constraints	Absolute structure parameter: 0.38 (12)

### Fractional atomic coordinates and isotropic or equivalent isotropic displacement parameters (Å^2^)

**Table:**

	*x*	*y*	*z*	*U*_iso_*/*U*_eq_	Occ. (<1)
Ge1	0	−1	0	0.0229 (6)	
Ge2	0.67726 (16)	0.0274 (3)	0.25	0.0139 (4)	0.978 (8)
Ge3	0.333333	−0.333333	−0.00011 (9)	0.0197 (5)	
Ni1	0.99243 (15)	0.32991 (12)	0.12498 (12)	0.0098 (3)	
Ni2	0.333333	−0.333333	0.25	0.0091 (5)	
Ni3	1	0	0.25	0.0197 (16)	0.508 (6)
Ni4	0.3335 (3)	−0.6665 (3)	0	0.0071 (8)	0.508 (10)

### Atomic displacement parameters (Å^2^)

**Table:**

	*U*^11^	*U*^22^	*U*^33^	*U*^12^	*U*^13^	*U*^23^
Ge1	0.0319 (8)	0.0319 (8)	0.0050 (9)	0.0160 (4)	0	0
Ge2	0.0136 (5)	0.0087 (4)	0.0053 (6)	−0.0051 (3)	0	0
Ge3	0.0267 (6)	0.0267 (6)	0.0058 (9)	0.0134 (3)	0	0
Ni1	0.0121 (4)	0.0128 (4)	0.0042 (4)	0.0060 (3)	0.0000 (2)	0.0004 (3)
Ni2	0.0110 (6)	0.0110 (6)	0.0053 (8)	0.0055 (3)	0	0
Ni3	0.0176 (17)	0.0176 (17)	0.024 (3)	0.0088 (8)	0	0
Ni4	0.0080 (8)	0.0080 (8)	0.0071 (14)	0.0055 (7)	−0.0006 (2)	0.0006 (2)

### Geometric parameters (Å, º)

**Table:**

Ge1—Ni1^i^	2.5473 (13)	Ge2—Ni4^xiv^	2.4955 (14)
Ge1—Ni1^ii^	2.5473 (15)	Ge2—Ni4^xv^	2.4955 (14)
Ge1—Ni1^iii^	2.5473 (16)	Ge3—Ni1^i^	2.5774 (15)
Ge1—Ni1^iv^	2.5473 (13)	Ge3—Ni1^ix^	2.5774 (14)
Ge1—Ni1^v^	2.5473 (15)	Ge3—Ni1^x^	2.5774 (15)
Ge1—Ni1^vi^	2.5473 (16)	Ge3—Ni1^xvi^	2.5104 (14)
Ge1—Ni3^i^	2.4904 (14)	Ge3—Ni1^xvii^	2.5104 (16)
Ge1—Ni3^iv^	2.4904 (14)	Ge3—Ni1^vi^	2.5104 (15)
Ge1—Ni4	2.2203 (18)	Ge3—Ni2	2.4915 (16)
Ge1—Ni4^vii^	2.220 (2)	Ge3—Ni4	2.219 (2)
Ge1—Ni4^viii^	2.220 (2)	Ge3—Ni4^xiv^	2.219 (2)
Ge2—Ni1	2.4053 (16)	Ge3—Ni4^iii^	2.219 (3)
Ge2—Ni1^ix^	2.709 (2)	Ni1—Ni1^xi^	2.491 (2)
Ge2—Ni1^x^	2.5275 (19)	Ni1—Ni1^xviii^	2.492 (2)
Ge2—Ni1^xi^	2.4053 (16)	Ni1—Ni2^xix^	2.5771 (14)
Ge2—Ni1^xii^	2.709 (2)	Ni1—Ni3	2.5476 (13)
Ge2—Ni1^xiii^	2.5275 (19)	Ni1—Ni4^xix^	2.579 (2)
Ge2—Ni2	2.3480 (17)	Ni1—Ni4^xiv^	2.5134 (19)
Ge2—Ni3	2.2457 (16)	Ni1—Ni4^xx^	2.542 (2)
			
Ni1^i^—Ge1—Ni1^ii^	98.15 (4)	Ge3^xix^—Ni1—Ni4^xx^	51.37 (6)
Ni1^i^—Ge1—Ni1^iii^	98.15 (4)	Ge3^xxii^—Ni1—Ni1^xi^	119.70 (5)
Ni1^i^—Ge1—Ni1^iv^	130.17 (3)	Ge3^xxii^—Ni1—Ni1^xviii^	62.03 (4)
Ni1^i^—Ge1—Ni1^v^	126.39 (4)	Ge3^xxii^—Ni1—Ni2^xix^	128.37 (4)
Ni1^i^—Ge1—Ni1^vi^	58.56 (4)	Ge3^xxii^—Ni1—Ni3	129.77 (3)
Ni1^i^—Ge1—Ni3^i^	60.74 (3)	Ge3^xxii^—Ni1—Ni4^xix^	121.43 (6)
Ni1^i^—Ge1—Ni3^iv^	119.26 (3)	Ge3^xxii^—Ni1—Ni4^xiv^	52.43 (6)
Ni1^i^—Ge1—Ni4	65.08 (5)	Ge3^xxii^—Ni1—Ni4^xx^	52.10 (5)
Ni1^i^—Ge1—Ni4^vii^	63.19 (5)	Ni1^xi^—Ni1—Ni1^xviii^	177.99 (5)
Ni1^i^—Ge1—Ni4^viii^	150.72 (3)	Ni1^xi^—Ni1—Ni2^xix^	61.10 (3)
Ni1^ii^—Ge1—Ni1^iii^	98.15 (4)	Ni1^xi^—Ni1—Ni3	60.73 (3)
Ni1^ii^—Ge1—Ni1^iv^	126.39 (4)	Ni1^xi^—Ni1—Ni4^xix^	118.86 (5)
Ni1^ii^—Ge1—Ni1^v^	58.56 (4)	Ni1^xi^—Ni1—Ni4^xiv^	119.69 (4)
Ni1^ii^—Ge1—Ni1^vi^	130.17 (4)	Ni1^xi^—Ni1—Ni4^xx^	119.32 (5)
Ni1^ii^—Ge1—Ni3^i^	60.74 (3)	Ni1^xviii^—Ni1—Ni2^xix^	117.14 (4)
Ni1^ii^—Ge1—Ni3^iv^	119.26 (3)	Ni1^xviii^—Ni1—Ni3	119.21 (5)
Ni1^ii^—Ge1—Ni4	150.72 (3)	Ni1^xviii^—Ni1—Ni4^xix^	59.40 (4)
Ni1^ii^—Ge1—Ni4^vii^	65.08 (6)	Ni1^xviii^—Ni1—Ni4^xiv^	62.04 (4)
Ni1^ii^—Ge1—Ni4^viii^	63.19 (6)	Ni1^xviii^—Ni1—Ni4^xx^	60.66 (5)
Ni1^iii^—Ge1—Ni1^iv^	58.56 (4)	Ni2^xix^—Ni1—Ni3	97.20 (4)
Ni1^iii^—Ge1—Ni1^v^	130.17 (4)	Ni2^xix^—Ni1—Ni4^xix^	80.61 (4)
Ni1^iii^—Ge1—Ni1^vi^	126.39 (3)	Ni2^xix^—Ni1—Ni4^xiv^	178.67 (7)
Ni1^iii^—Ge1—Ni3^i^	60.74 (3)	Ni2^xix^—Ni1—Ni4^xx^	81.32 (4)
Ni1^iii^—Ge1—Ni3^iv^	119.26 (3)	Ni3—Ni1—Ni4^xix^	81.20 (5)
Ni1^iii^—Ge1—Ni4	63.19 (5)	Ni3—Ni1—Ni4^xiv^	82.48 (6)
Ni1^iii^—Ge1—Ni4^vii^	150.72 (3)	Ni3—Ni1—Ni4^xx^	178.03 (6)
Ni1^iii^—Ge1—Ni4^viii^	65.08 (5)	Ni4^xix^—Ni1—Ni4^xiv^	98.06 (6)
Ni1^iv^—Ge1—Ni1^v^	98.15 (4)	Ni4^xix^—Ni1—Ni4^xx^	97.26 (7)
Ni1^iv^—Ge1—Ni1^vi^	98.15 (4)	Ni4^xiv^—Ni1—Ni4^xx^	98.97 (7)
Ni1^iv^—Ge1—Ni3^i^	119.26 (3)	Ge2—Ni2—Ge2^xiv^	120.00 (6)
Ni1^iv^—Ge1—Ni3^iv^	60.74 (3)	Ge2—Ni2—Ge2^iii^	120.00 (6)
Ni1^iv^—Ge1—Ni4	65.08 (5)	Ge2—Ni2—Ge3	90
Ni1^iv^—Ge1—Ni4^vii^	150.72 (3)	Ge2—Ni2—Ge3^xi^	90
Ni1^iv^—Ge1—Ni4^viii^	63.19 (5)	Ge2—Ni2—Ni1^i^	150.96 (3)
Ni1^v^—Ge1—Ni1^vi^	98.15 (4)	Ge2—Ni2—Ni1^ix^	66.56 (4)
Ni1^v^—Ge1—Ni3^i^	119.26 (3)	Ge2—Ni2—Ni1^x^	61.54 (4)
Ni1^v^—Ge1—Ni3^iv^	60.74 (3)	Ge2—Ni2—Ni1^xxiii^	150.96 (3)
Ni1^v^—Ge1—Ni4	150.72 (3)	Ge2—Ni2—Ni1^xii^	66.56 (4)
Ni1^v^—Ge1—Ni4^vii^	63.19 (6)	Ge2—Ni2—Ni1^xiii^	61.54 (4)
Ni1^v^—Ge1—Ni4^viii^	65.08 (6)	Ge2^xiv^—Ni2—Ge2^iii^	120.00 (6)
Ni1^vi^—Ge1—Ni3^i^	119.26 (3)	Ge2^xiv^—Ni2—Ge3	90
Ni1^vi^—Ge1—Ni3^iv^	60.74 (3)	Ge2^xiv^—Ni2—Ge3^xi^	90
Ni1^vi^—Ge1—Ni4	63.19 (5)	Ge2^xiv^—Ni2—Ni1^i^	61.54 (5)
Ni1^vi^—Ge1—Ni4^vii^	65.08 (5)	Ge2^xiv^—Ni2—Ni1^ix^	150.96 (3)
Ni1^vi^—Ge1—Ni4^viii^	150.72 (3)	Ge2^xiv^—Ni2—Ni1^x^	66.56 (5)
Ni3^i^—Ge1—Ni3^iv^	180.0 (5)	Ge2^xiv^—Ni2—Ni1^xxiii^	61.54 (5)
Ni3^i^—Ge1—Ni4	90	Ge2^xiv^—Ni2—Ni1^xii^	150.96 (3)
Ni3^i^—Ge1—Ni4^vii^	90	Ge2^xiv^—Ni2—Ni1^xiii^	66.56 (5)
Ni3^i^—Ge1—Ni4^viii^	90	Ge2^iii^—Ni2—Ge3	90
Ni3^iv^—Ge1—Ni4	90	Ge2^iii^—Ni2—Ge3^xi^	90
Ni3^iv^—Ge1—Ni4^vii^	90	Ge2^iii^—Ni2—Ni1^i^	66.56 (5)
Ni3^iv^—Ge1—Ni4^viii^	90	Ge2^iii^—Ni2—Ni1^ix^	61.54 (4)
Ni4—Ge1—Ni4^vii^	120.00 (8)	Ge2^iii^—Ni2—Ni1^x^	150.96 (3)
Ni4—Ge1—Ni4^viii^	120.00 (8)	Ge2^iii^—Ni2—Ni1^xxiii^	66.56 (5)
Ni4^vii^—Ge1—Ni4^viii^	120.00 (8)	Ge2^iii^—Ni2—Ni1^xii^	61.54 (4)
Ni1—Ge2—Ni1^ix^	97.46 (5)	Ge2^iii^—Ni2—Ni1^xiii^	150.96 (3)
Ni1—Ge2—Ni1^x^	99.90 (6)	Ge3—Ni2—Ge3^xi^	180.0 (5)
Ni1—Ge2—Ni1^xi^	62.37 (5)	Ge3—Ni2—Ni1^i^	61.10 (3)
Ni1—Ge2—Ni1^xii^	127.29 (7)	Ge3—Ni2—Ni1^ix^	61.10 (3)
Ni1—Ge2—Ni1^xiii^	133.02 (8)	Ge3—Ni2—Ni1^x^	61.10 (3)
Ni1—Ge2—Ni2	148.54 (3)	Ge3—Ni2—Ni1^xxiii^	118.90 (3)
Ni1—Ge2—Ni3	66.32 (4)	Ge3—Ni2—Ni1^xii^	118.90 (3)
Ni1—Ge2—Ni4^xiv^	61.68 (5)	Ge3—Ni2—Ni1^xiii^	118.90 (3)
Ni1—Ge2—Ni4^xv^	123.99 (5)	Ge3^xi^—Ni2—Ni1^i^	118.90 (3)
Ni1^ix^—Ge2—Ni1^x^	96.47 (4)	Ge3^xi^—Ni2—Ni1^ix^	118.90 (3)
Ni1^ix^—Ge2—Ni1^xi^	127.29 (7)	Ge3^xi^—Ni2—Ni1^x^	118.90 (3)
Ni1^ix^—Ge2—Ni1^xii^	54.74 (5)	Ge3^xi^—Ni2—Ni1^xxiii^	61.10 (3)
Ni1^ix^—Ge2—Ni1^xiii^	124.45 (5)	Ge3^xi^—Ni2—Ni1^xii^	61.10 (3)
Ni1^ix^—Ge2—Ni2	60.78 (4)	Ge3^xi^—Ni2—Ni1^xiii^	61.10 (3)
Ni1^ix^—Ge2—Ni3	61.03 (4)	Ni1^i^—Ni2—Ni1^ix^	98.61 (4)
Ni1^ix^—Ge2—Ni4^xiv^	59.25 (7)	Ni1^i^—Ni2—Ni1^x^	98.61 (4)
Ni1^ix^—Ge2—Ni4^xv^	113.96 (9)	Ni1^i^—Ni2—Ni1^xxiii^	57.80 (4)
Ni1^x^—Ge2—Ni1^xi^	133.02 (8)	Ni1^i^—Ni2—Ni1^xii^	128.08 (4)
Ni1^x^—Ge2—Ni1^xii^	124.45 (5)	Ni1^i^—Ni2—Ni1^xiii^	128.08 (4)
Ni1^x^—Ge2—Ni1^xiii^	59.04 (5)	Ni1^ix^—Ni2—Ni1^x^	98.61 (4)
Ni1^x^—Ge2—Ni2	63.70 (3)	Ni1^ix^—Ni2—Ni1^xxiii^	128.08 (4)
Ni1^x^—Ge2—Ni3	149.73 (4)	Ni1^ix^—Ni2—Ni1^xii^	57.80 (4)
Ni1^x^—Ge2—Ni4^xiv^	60.81 (6)	Ni1^ix^—Ni2—Ni1^xiii^	128.08 (3)
Ni1^x^—Ge2—Ni4^xv^	119.72 (7)	Ni1^x^—Ni2—Ni1^xxiii^	128.08 (4)
Ni1^xi^—Ge2—Ni1^xii^	97.46 (5)	Ni1^x^—Ni2—Ni1^xii^	128.08 (3)
Ni1^xi^—Ge2—Ni1^xiii^	99.90 (6)	Ni1^x^—Ni2—Ni1^xiii^	57.80 (4)
Ni1^xi^—Ge2—Ni2	148.54 (3)	Ni1^xxiii^—Ni2—Ni1^xii^	98.61 (4)
Ni1^xi^—Ge2—Ni3	66.32 (4)	Ni1^xxiii^—Ni2—Ni1^xiii^	98.61 (4)
Ni1^xi^—Ge2—Ni4^xiv^	123.99 (5)	Ni1^xii^—Ni2—Ni1^xiii^	98.61 (4)
Ni1^xi^—Ge2—Ni4^xv^	61.68 (5)	Ge1^xix^—Ni3—Ge1^xxiv^	180.0 (5)
Ni1^xii^—Ge2—Ni1^xiii^	96.47 (4)	Ge1^xix^—Ni3—Ge2	90
Ni1^xii^—Ge2—Ni2	60.78 (4)	Ge1^xix^—Ni3—Ge2^ix^	90
Ni1^xii^—Ge2—Ni3	61.03 (4)	Ge1^xix^—Ni3—Ge2^xx^	90
Ni1^xii^—Ge2—Ni4^xiv^	113.96 (9)	Ge1^xix^—Ni3—Ni1	60.73 (3)
Ni1^xii^—Ge2—Ni4^xv^	59.25 (7)	Ge1^xix^—Ni3—Ni1^ix^	60.73 (3)
Ni1^xiii^—Ge2—Ni2	63.70 (3)	Ge1^xix^—Ni3—Ni1^xx^	60.73 (3)
Ni1^xiii^—Ge2—Ni3	149.73 (4)	Ge1^xix^—Ni3—Ni1^xi^	119.27 (3)
Ni1^xiii^—Ge2—Ni4^xiv^	119.72 (7)	Ge1^xix^—Ni3—Ni1^xii^	119.27 (3)
Ni1^xiii^—Ge2—Ni4^xv^	60.81 (6)	Ge1^xix^—Ni3—Ni1^xxv^	119.27 (3)
Ni2—Ge2—Ni3	113.60 (8)	Ge1^xxiv^—Ni3—Ge2	90
Ni2—Ge2—Ni4^xiv^	87.00 (4)	Ge1^xxiv^—Ni3—Ge2^ix^	90
Ni2—Ge2—Ni4^xv^	87.00 (4)	Ge1^xxiv^—Ni3—Ge2^xx^	90
Ni3—Ge2—Ni4^xiv^	89.29 (6)	Ge1^xxiv^—Ni3—Ni1	119.27 (3)
Ni3—Ge2—Ni4^xv^	89.29 (6)	Ge1^xxiv^—Ni3—Ni1^ix^	119.27 (3)
Ni4^xiv^—Ge2—Ni4^xv^	172.68 (10)	Ge1^xxiv^—Ni3—Ni1^xx^	119.27 (3)
Ni1^i^—Ge3—Ni1^ix^	98.59 (4)	Ge1^xxiv^—Ni3—Ni1^xi^	60.73 (3)
Ni1^i^—Ge3—Ni1^x^	98.59 (4)	Ge1^xxiv^—Ni3—Ni1^xii^	60.73 (3)
Ni1^i^—Ge3—Ni1^xvi^	127.35 (3)	Ge1^xxiv^—Ni3—Ni1^xxv^	60.73 (3)
Ni1^i^—Ge3—Ni1^xvii^	129.24 (3)	Ge2—Ni3—Ge2^ix^	120.00 (5)
Ni1^i^—Ge3—Ni1^vi^	58.62 (4)	Ge2—Ni3—Ge2^xx^	120.00 (6)
Ni1^i^—Ge3—Ni2	61.09 (3)	Ge2—Ni3—Ni1	59.84 (4)
Ni1^i^—Ge3—Ni4	64.56 (4)	Ge2—Ni3—Ni1^ix^	68.50 (4)
Ni1^i^—Ge3—Ni4^xiv^	151.06 (5)	Ge2—Ni3—Ni1^xx^	150.32 (3)
Ni1^i^—Ge3—Ni4^iii^	63.50 (4)	Ge2—Ni3—Ni1^xi^	59.84 (4)
Ni1^ix^—Ge3—Ni1^x^	98.59 (5)	Ge2—Ni3—Ni1^xii^	68.50 (4)
Ni1^ix^—Ge3—Ni1^xvi^	129.24 (3)	Ge2—Ni3—Ni1^xxv^	150.32 (3)
Ni1^ix^—Ge3—Ni1^xvii^	58.62 (4)	Ge2^ix^—Ni3—Ge2^xx^	120.00 (6)
Ni1^ix^—Ge3—Ni1^vi^	127.35 (4)	Ge2^ix^—Ni3—Ni1	150.32 (3)
Ni1^ix^—Ge3—Ni2	61.09 (3)	Ge2^ix^—Ni3—Ni1^ix^	59.84 (5)
Ni1^ix^—Ge3—Ni4	63.50 (4)	Ge2^ix^—Ni3—Ni1^xx^	68.50 (4)
Ni1^ix^—Ge3—Ni4^xiv^	64.56 (6)	Ge2^ix^—Ni3—Ni1^xi^	150.32 (3)
Ni1^ix^—Ge3—Ni4^iii^	151.06 (5)	Ge2^ix^—Ni3—Ni1^xii^	59.84 (5)
Ni1^x^—Ge3—Ni1^xvi^	58.62 (4)	Ge2^ix^—Ni3—Ni1^xxv^	68.50 (4)
Ni1^x^—Ge3—Ni1^xvii^	127.35 (3)	Ge2^xx^—Ni3—Ni1	68.50 (5)
Ni1^x^—Ge3—Ni1^vi^	129.24 (4)	Ge2^xx^—Ni3—Ni1^ix^	150.32 (3)
Ni1^x^—Ge3—Ni2	61.09 (3)	Ge2^xx^—Ni3—Ni1^xx^	59.84 (5)
Ni1^x^—Ge3—Ni4	151.06 (5)	Ge2^xx^—Ni3—Ni1^xi^	68.50 (5)
Ni1^x^—Ge3—Ni4^xiv^	63.50 (6)	Ge2^xx^—Ni3—Ni1^xii^	150.32 (3)
Ni1^x^—Ge3—Ni4^iii^	64.56 (4)	Ge2^xx^—Ni3—Ni1^xxv^	59.84 (5)
Ni1^xvi^—Ge3—Ni1^xvii^	97.57 (4)	Ni1—Ni3—Ni1^ix^	98.13 (4)
Ni1^xvi^—Ge3—Ni1^vi^	97.57 (5)	Ni1—Ni3—Ni1^xx^	98.13 (4)
Ni1^xvi^—Ge3—Ni2	119.70 (3)	Ni1—Ni3—Ni1^xi^	58.53 (4)
Ni1^xvi^—Ge3—Ni4	150.32 (5)	Ni1—Ni3—Ni1^xii^	128.28 (4)
Ni1^xvi^—Ge3—Ni4^xiv^	64.69 (6)	Ni1—Ni3—Ni1^xxv^	128.28 (3)
Ni1^xvi^—Ge3—Ni4^iii^	63.86 (4)	Ni1^ix^—Ni3—Ni1^xx^	98.13 (4)
Ni1^xvii^—Ge3—Ni1^vi^	97.57 (5)	Ni1^ix^—Ni3—Ni1^xi^	128.28 (4)
Ni1^xvii^—Ge3—Ni2	119.70 (3)	Ni1^ix^—Ni3—Ni1^xii^	58.53 (4)
Ni1^xvii^—Ge3—Ni4	64.69 (4)	Ni1^ix^—Ni3—Ni1^xxv^	128.28 (4)
Ni1^xvii^—Ge3—Ni4^xiv^	63.86 (6)	Ni1^xx^—Ni3—Ni1^xi^	128.28 (3)
Ni1^xvii^—Ge3—Ni4^iii^	150.32 (5)	Ni1^xx^—Ni3—Ni1^xii^	128.28 (4)
Ni1^vi^—Ge3—Ni2	119.70 (3)	Ni1^xx^—Ni3—Ni1^xxv^	58.53 (4)
Ni1^vi^—Ge3—Ni4	63.86 (5)	Ni1^xi^—Ni3—Ni1^xii^	98.13 (4)
Ni1^vi^—Ge3—Ni4^xiv^	150.32 (5)	Ni1^xi^—Ni3—Ni1^xxv^	98.13 (4)
Ni1^vi^—Ge3—Ni4^iii^	64.69 (5)	Ni1^xii^—Ni3—Ni1^xxv^	98.13 (4)
Ni2—Ge3—Ni4	89.97 (2)	Ge1—Ni4—Ge2^iii^	90.46 (4)
Ni2—Ge3—Ni4^xiv^	89.97 (2)	Ge1—Ni4—Ge2^vi^	90.46 (4)
Ni2—Ge3—Ni4^iii^	89.97 (2)	Ge1—Ni4—Ge3	119.98 (9)
Ni4—Ge3—Ni4^xiv^	120.00 (8)	Ge1—Ni4—Ge3^xxvi^	119.98 (9)
Ni4—Ge3—Ni4^iii^	120.00 (6)	Ge1—Ni4—Ni1^i^	63.59 (4)
Ni4^xiv^—Ge3—Ni4^iii^	120.00 (8)	Ge1—Ni4—Ni1^ix^	150.66 (3)
Ge1^xix^—Ni1—Ge2	85.18 (5)	Ge1—Ni4—Ni1^iii^	64.77 (5)
Ge1^xix^—Ni1—Ge2^xxi^	178.36 (5)	Ge1—Ni4—Ni1^iv^	63.59 (4)
Ge1^xix^—Ni1—Ge2^xx^	79.21 (4)	Ge1—Ni4—Ni1^xvii^	150.66 (3)
Ge1^xix^—Ni1—Ge3^xix^	97.20 (4)	Ge1—Ni4—Ni1^vi^	64.77 (5)
Ge1^xix^—Ni1—Ge3^xxii^	98.94 (4)	Ge2^iii^—Ni4—Ge2^vi^	179.09 (7)
Ge1^xix^—Ni1—Ni1^xi^	119.26 (5)	Ge2^iii^—Ni4—Ge3	92.94 (6)
Ge1^xix^—Ni1—Ni1^xviii^	60.72 (4)	Ge2^iii^—Ni4—Ge3^xxvi^	86.60 (7)
Ge1^xix^—Ni1—Ni2^xix^	126.72 (4)	Ge2^iii^—Ni4—Ni1^i^	64.51 (6)
Ge1^xix^—Ni1—Ni3	58.53 (3)	Ge2^iii^—Ni4—Ni1^ix^	60.22 (4)
Ge1^xix^—Ni1—Ni4^xix^	51.32 (4)	Ge2^iii^—Ni4—Ni1^iii^	57.40 (5)
Ge1^xix^—Ni1—Ni4^xiv^	52.04 (6)	Ge2^iii^—Ni4—Ni1^iv^	115.94 (8)
Ge1^xix^—Ni1—Ni4^xx^	121.38 (5)	Ge2^iii^—Ni4—Ni1^xvii^	118.87 (6)
Ge2—Ni1—Ge2^xxi^	93.40 (6)	Ge2^iii^—Ni4—Ni1^vi^	123.07 (9)
Ge2—Ni1—Ge2^xx^	98.85 (5)	Ge2^vi^—Ni4—Ge3	86.60 (7)
Ge2—Ni1—Ge3^xix^	177.41 (6)	Ge2^vi^—Ni4—Ge3^xxvi^	92.94 (6)
Ge2—Ni1—Ge3^xxii^	82.42 (5)	Ge2^vi^—Ni4—Ni1^i^	115.94 (8)
Ge2—Ni1—Ni1^xi^	58.82 (3)	Ge2^vi^—Ni4—Ni1^ix^	118.87 (6)
Ge2—Ni1—Ni1^xviii^	122.95 (4)	Ge2^vi^—Ni4—Ni1^iii^	123.07 (9)
Ge2—Ni1—Ni2^xix^	119.90 (5)	Ge2^vi^—Ni4—Ni1^iv^	64.51 (6)
Ge2—Ni1—Ni3	53.83 (4)	Ge2^vi^—Ni4—Ni1^xvii^	60.22 (4)
Ge2—Ni1—Ni4^xix^	130.77 (7)	Ge2^vi^—Ni4—Ni1^vi^	57.40 (5)
Ge2—Ni1—Ni4^xiv^	60.93 (3)	Ge3—Ni4—Ge3^xxvi^	120.04 (6)
Ge2—Ni1—Ni4^xx^	128.05 (8)	Ge3—Ni4—Ni1^i^	64.47 (6)
Ge2^xxi^—Ni1—Ge2^xx^	101.84 (5)	Ge3—Ni4—Ni1^ix^	65.13 (5)
Ge2^xxi^—Ni1—Ge3^xix^	84.23 (4)	Ge3—Ni4—Ni1^iii^	150.33 (4)
Ge2^xxi^—Ni1—Ge3^xxii^	80.04 (5)	Ge3—Ni4—Ni1^iv^	151.11 (4)
Ge2^xxi^—Ni1—Ni1^xi^	60.48 (4)	Ge3—Ni4—Ni1^xvii^	63.21 (5)
Ge2^xxi^—Ni1—Ni1^xviii^	119.61 (6)	Ge3—Ni4—Ni1^vi^	63.72 (6)
Ge2^xxi^—Ni1—Ni2^xix^	54.76 (4)	Ge3^xxvi^—Ni4—Ni1^i^	151.11 (4)
Ge2^xxi^—Ni1—Ni3	121.18 (5)	Ge3^xxvi^—Ni4—Ni1^ix^	63.21 (5)
Ge2^xxi^—Ni1—Ni4^xix^	130.31 (5)	Ge3^xxvi^—Ni4—Ni1^iii^	63.72 (6)
Ge2^xxi^—Ni1—Ni4^xiv^	126.49 (8)	Ge3^xxvi^—Ni4—Ni1^iv^	64.47 (6)
Ge2^xxi^—Ni1—Ni4^xx^	58.97 (4)	Ge3^xxvi^—Ni4—Ni1^xvii^	65.13 (5)
Ge2^xx^—Ni1—Ge3^xix^	80.68 (4)	Ge3^xxvi^—Ni4—Ni1^vi^	150.33 (4)
Ge2^xx^—Ni1—Ge3^xxii^	177.63 (6)	Ni1^i^—Ni4—Ni1^ix^	99.46 (6)
Ge2^xx^—Ni1—Ni1^xi^	62.63 (4)	Ni1^i^—Ni4—Ni1^iii^	98.19 (5)
Ge2^xx^—Ni1—Ni1^xviii^	115.64 (5)	Ni1^i^—Ni4—Ni1^iv^	127.19 (6)
Ge2^xx^—Ni1—Ni2^xix^	52.67 (3)	Ni1^i^—Ni4—Ni1^xvii^	127.67 (9)
Ge2^xx^—Ni1—Ni3	50.47 (3)	Ni1^i^—Ni4—Ni1^vi^	58.56 (6)
Ge2^xx^—Ni1—Ni4^xix^	56.25 (4)	Ni1^ix^—Ni4—Ni1^iii^	96.67 (6)
Ge2^xx^—Ni1—Ni4^xiv^	126.49 (7)	Ni1^ix^—Ni4—Ni1^iv^	127.67 (9)
Ge2^xx^—Ni1—Ni4^xx^	127.63 (6)	Ni1^ix^—Ni4—Ni1^xvii^	58.68 (5)
Ge3^xix^—Ni1—Ge3^xxii^	98.14 (4)	Ni1^ix^—Ni4—Ni1^vi^	128.84 (9)
Ge3^xix^—Ni1—Ni1^xi^	118.91 (4)	Ni1^iii^—Ni4—Ni1^iv^	58.56 (6)
Ge3^xix^—Ni1—Ni1^xviii^	59.34 (4)	Ni1^iii^—Ni4—Ni1^xvii^	128.84 (9)
Ge3^xix^—Ni1—Ni2^xix^	57.81 (3)	Ni1^iii^—Ni4—Ni1^vi^	129.53 (6)
Ge3^xix^—Ni1—Ni3	126.73 (5)	Ni1^iv^—Ni4—Ni1^xvii^	99.46 (6)
Ge3^xix^—Ni1—Ni4^xix^	50.98 (4)	Ni1^iv^—Ni4—Ni1^vi^	98.19 (5)
Ge3^xix^—Ni1—Ni4^xiv^	121.39 (6)	Ni1^xvii^—Ni4—Ni1^vi^	96.67 (6)

Symmetry codes: (i) *x*−1, *y*−1, *z*; (ii) −*y*, *x*−*y*−2, *z*; (iii) −*x*+*y*+1, −*x*, *z*; (iv) *y*, *x*−2, −*z*; (v) *x*−*y*−1, −*y*−1, −*z*; (vi) −*x*+1, −*x*+*y*, −*z*; (vii) −*y*−1, *x*−*y*−2, *z*; (viii) −*x*+*y*+1, −*x*−1, *z*; (ix) −*y*+1, *x*−*y*−1, *z*; (x) −*x*+*y*+1, −*x*+1, *z*; (xi) *x*, *y*, −*z*+1/2; (xii) −*y*+1, *x*−*y*−1, −*z*+1/2; (xiii) −*x*+*y*+1, −*x*+1, −*z*+1/2; (xiv) −*y*, *x*−*y*−1, *z*; (xv) −*y*, *x*−*y*−1, −*z*+1/2; (xvi) *y*, *x*−1, −*z*; (xvii) *x*−*y*, −*y*, −*z*; (xviii) −*x*+2, −*x*+*y*+1, −*z*; (xix) *x*+1, *y*+1, *z*; (xx) −*x*+*y*+2, −*x*+1, *z*; (xxi) −*y*+1, *x*−*y*, *z*; (xxii) *y*+1, *x*, −*z*; (xxiii) *x*−1, *y*−1, −*z*+1/2; (xxiv) *x*+1, *y*+1, −*z*+1/2; (xxv) −*x*+*y*+2, −*x*+1, −*z*+1/2; (xxvi) *y*+1, *x*−1, −*z*.
